# Analyzing the Complex Regulatory Landscape of Hfq – an Integrative, Multi-Omics Approach

**DOI:** 10.3389/fmicb.2017.01784

**Published:** 2017-09-20

**Authors:** Lucia Grenga, Govind Chandra, Gerhard Saalbach, Carla V. Galmozzi, Günter Kramer, Jacob G. Malone

**Affiliations:** ^1^Department of Molecular Microbiology, John Innes Centre Norwich, United Kingdom; ^2^School of Biological Sciences, University of East Anglia Norwich, United Kingdom; ^3^Center for Molecular Biology of the University of Heidelberg, DKFZ-ZMBH Alliance Heidelberg, Germany; ^4^German Cancer Research Center Heidelberg, Germany

**Keywords:** *Pseudomonas*, ribosomal profiling, multi-omics analysis, Hfq, integrative approach

## Abstract

The ability of bacteria to respond to environmental change is based on the ability to coordinate, redirect and fine-tune their genetic repertoire as and when required. While we can learn a great deal from reductive analysis of individual pathways and global approaches to gene regulation, a deeper understanding of these complex signaling networks requires the simultaneous consideration of several regulatory layers at the genome scale. To highlight the power of this approach we analyzed the Hfq transcriptional/translational regulatory network in the model bacterium *Pseudomonas fluorescens*. We first used extensive ‘omics’ analyses to assess how *hfq* deletion affects mRNA abundance, mRNA translation and protein abundance. The subsequent, multi-level integration of these datasets allows us to highlight the discrete contributions by Hfq to gene regulation at different levels. The integrative approach to regulatory analysis we describe here has significant potential, for both dissecting individual signaling pathways and understanding the strategies bacteria use to cope with external challenges.

## Introduction

The ability to control expression of their genetic repertoire is a key strategy adopted by bacteria to adapt to changing environments, and cope with a range of external challenges (Fong et al., 2005; López-Maury et al., 2008; Silva et al., 2011). However, transcriptional control does not generally occur independently of other regulatory mechanisms. While regulation at the level of transcript abundance represents an important level of control, it is emerging that the extent of post-transcriptional regulation of gene expression in bacteria has been relatively underestimated, with recent studies highlighting the central importance of integrated post-transcriptional and post-translational control mechanisms to the formation of a plastic environmental response (Picard et al., 2009; Jeong et al., 2016). To fully understand how global regulatory responses are controlled requires the genome-scale characterization of several layers of regulation, including both post-transcriptional and post-translational events, and the integration of multiple ‘omics’ analyses.

To illustrate the relevance of this approach we focused on the protein Hfq in the plant growth-promoting bacterium *Pseudomonas fluorescens*. Hfq is a pleiotropic regulator of bacterial gene expression impacting, in some organisms, the expression of up to 20% of all genes (Chao and Vogel, 2010). The regulatory role of Hfq is ascribed mainly to its function as an RNA-chaperone, facilitating interactions between bacterial non-coding RNAs and their mRNA targets. Thus, Hfq modulates mRNA stability and translation alongside sRNA-mediated transcription antitermination (Sedlyarova et al., 2016). Importantly, Hfq can also regulate gene expression by acting alone and influencing mRNA polyadenylation or translation (Valentin-Hansen et al., 2004), or by directly interacting with DNA (Cech et al., 2016). In *P. fluorescens*, Hfq is not essential for survival but plays a critical role during niche adaptation, with reduced Hfq levels resulting in pronounced proteomic changes and phenotypes including increased surface attachment, reduced motility and severely compromised wheat rhizosphere colonization (Little et al., 2016).

Several studies have been performed to characterize potential roles for Hfq in different bacteria by phenotypic, transcriptomic and proteomic analyses of deletion mutants (Sonnleitner et al., 2006; Torres-Quesada et al., 2010; Cui et al., 2013; Wilf et al., 2013; Bilusic et al., 2014; Boudry et al., 2014; Deng et al., 2014; Hämmerle et al., 2014; Holmqvist et al., 2016). However, the existence of different, interacting regulatory layers markedly reduces the predictive power of individual transcriptomic and proteomic studies (Arraiano and Maquat, 2003). For example, a purely transcriptomic approach to characterize Hfq-based regulation is likely to miss many important post-transcriptional interactions. Although measuring changes in protein abundance will capture translational impacts that occur without corresponding changes at the transcriptional level, such proteomic experiments are unable to distinguish between different levels of regulation. In addition, comprehensive proteomic analysis relies on state-of-the-art MS combined with accurate quantification methods.

Recently, RNomics and deep sequencing-led approaches to detect transcriptome-wide binding sites of Hfq in different bacteria have enabled researchers to examine the specificity of Hfq interactions with its RNA ligands (Feng et al., 2015; Papenfort et al., 2015; Holmqvist et al., 2016). Nonetheless, and despite these ground breaking analyses, many aspects of Hfq regulation remain unknown. Moreover, none of these studies shed light on the Hfq translatome, despite the importance of Hfq (alongside CsrA/RsmA and ProQ (Holmqvist et al., 2016; Smirnov et al., 2016) as a global regulator of post-transcriptional gene expression.

To address the complex role of Hfq in *P. fluorescens* and build a comprehensive model of its regulon, we carried out an extensive multi-omics (mRNA abundance, translatome and proteome) analysis of the *P. fluorescens* Δ*hfq* mutant. By combining datasets from three distinct experimental approaches, we are able to identify and dissect the effect of *hfq* deletion on gene regulation at different levels. Our analysis also provides evidence suggesting a novel role for Hfq as a non-specific regulator of ribosomal-RNA interaction. The workflow we describe here has enabled us to produce a highly comprehensive picture of bacterial gene regulation.

## Results

### Parallel Global Analyses of the *P. fluorescens* Δ*hfq* Mutant

To detect Hfq-regulated genes in *P. fluorescens* SBW25, we determined the transcriptomic, translatomic and proteomic profiles of the wild type and Δ*hfq* mutant strains (Supplementary Figure S1). First, to confirm that the Δ*hfq* mutant (Little et al., 2016) was non-polar we conducted qRT-PCR analysis on the downstream gene (*hflX*), whose expression was unaffected by *hfq* deletion. In addition, we were able to complement the *hfq* deletion phenotype with a plasmid-borne copy of *hfq* (Supplementary Figure S2). In each case, the experiments were carried out in identical conditions, with cells grown to late exponential phase in defined M9 medium supplemented with 0.4% pyruvate and 0.4% casamino acids. In this medium, compared to LB, the *hfq* mutant exhibited a reduced growth rate and entered stationary phase at a slightly lower cell density than wild type SBW25 (Supplementary Figure S2). This suggests that while *hfq* is not essential, it is required for optimal bacterial growth in *P. fluorescens.* Appropriate cell densities were then chosen to ensure that samples were taken from wild type and Δ*hfq* at comparable growth phases. We decided to characterize Hfq-mediated regulation in the late exponential phase, as this allowed us to avoid the drastic changes in gene expression patterns (both transcriptional and translational) that are often associated with entry into stationary phase. qRT-PCR performed on late exponential and stationary phase SBW25 cultures demonstrated that *hfq* transcription remains stable during this growth period (Supplementary Figure S2). Similarly, Western blotting with a C-terminal flag-tagged protein showed that Hfq abundance does not change substantially during this part of the SBW25 growth cycle (Supplementary Figure S2). Two biological replicates of each strain were analyzed for each dataset.

**FIGURE 1:**
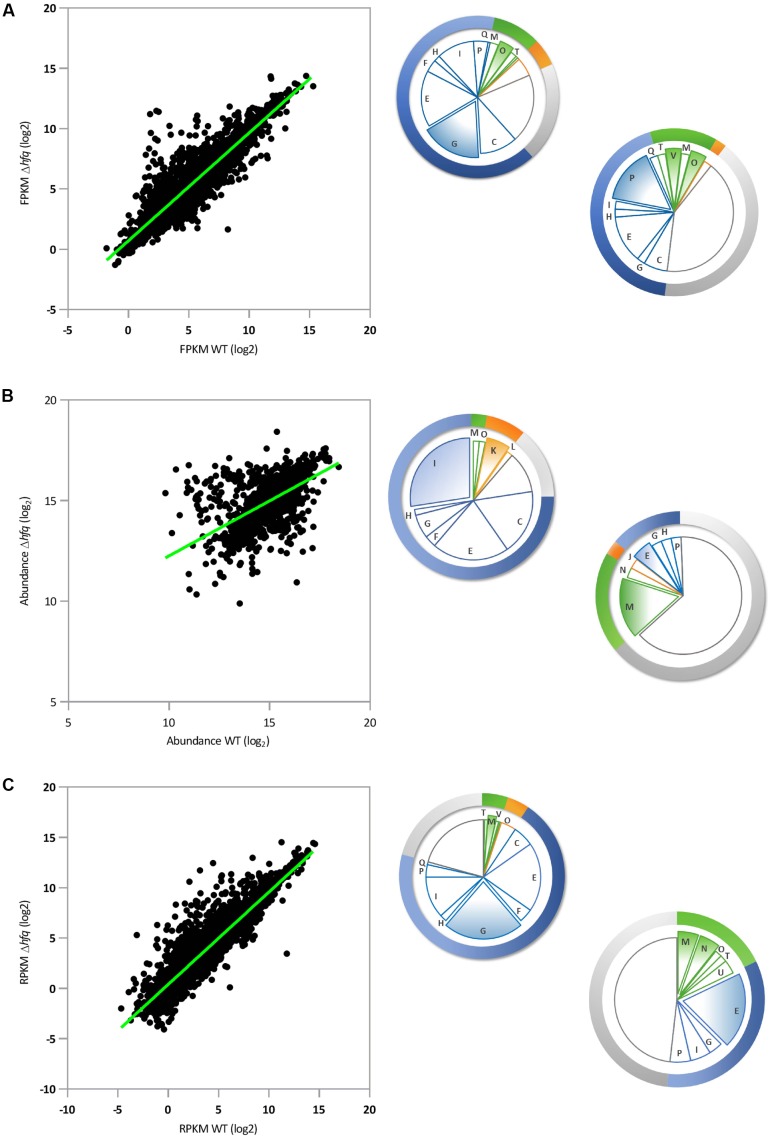
Parallel global analyses of the *P. fluorescens* Δ*hfq* mutant. **(A)** Transcriptomic analysis: Scatter-plot representing pairwise comparison of mean log_2_ FPKM expression values for *P. fluorescens* WT and Δ*hfq* (*n* = 5910). **(B)** Proteomic analysis: Scatter-plot representing pairwise comparison of mean log_2_ protein abundance values for *P. fluorescens* WT and Δ*hfq* (*n* = 2019). **(C)** Translatomic analysis: Scatter-plot representing pairwise comparison of mean log_2_ RPKM expression values for *P. fluorescens* WT and Δ*hfq* (*n* = 5910). The pie chart sections indicate the proportion of significantly up- (top left) or down- (bottom right) regulated genes in the Δ*hfq* background (according to the COG database) in each functional category. Categories are color-coded as follows: blue – metabolism, green – cellular processes and signaling, orange – information storage and processing, and gray – poorly characterized. The letters in each section of the chart refer to the respective COG functional categories. The most abundant categories are expanded from the chart in each case. A complete list of genes and information on their predicted functions are given in **Supplementary Table S1**.

In our RNA-Seq analysis, 212 mRNAs (out of 5910; *p*-value ≤ 0.01) were identified that showed statistically significant changes (log_2_FC = 2) between SBW25 WT and Δ*hfq*. A scatter plot of these *loci* comparing FPKM (Fragments Per Kilobase Million) expression values for WT and Δ*hfq* suggests that Hfq exerts a predominantly negative regulatory effect on transcript levels. Under the conditions tested, 46 mRNA were down regulated and 166 were up regulated compared to wild type, equivalent to 3.6% of all *P. fluorescens* genes (**Figure 1A**). Classification of Hfq-controlled mRNA according to COG database searches revealed that the deletion of *hfq* disproportionately affects the steady state transcript levels of genes involved in bacterial metabolism, with 128 mRNAs misregulated in the *hfq* mutant (60.3% of regulated mRNAs) as opposed to 10 involved in information storage and processing, 22 in cell processes and signaling, and 52 poorly characterized *loci*. Further subdivision of these categories revealed that the most abundant functional classes up regulated in the *Δhfq* background (i.e., negatively affected by Hfq) are involved in amino acid and carbohydrate transport and metabolism (**Figure 1A**). Conversely, the transcripts downregulated in the *Δhfq* mutant were most frequently associated with inorganic ion transport and metabolism (**Figure 1A**). These findings were supported by GO enrichment analysis of the RNA-Seq data (**Supplementary Table S4**).

We then purified the soluble proteomes of both strains under the same experimental conditions, and quantitatively analyzed them using isobaric labeling (iTRAQ). Following the iTRAQ analysis, we focussed our attention on the 1874 proteins identified in both WT and *Δhfq* lysates (with at least 3 abundance counts, *p*-value ≤ 0.01, and FDR 1%) and representing 66% of the total predicted non-membrane-associated proteome. (This sample represents a substantial fraction of the total cellular proteome, albeit one missing most membrane-associated proteins.) A scatter plot comparing the proteomic profiles of WT and *Δhfq* (**Figure 1B**) revealed that 99 proteins (5.3% of the total identified proteome) were differentially present in the *hfq* null mutant compared to the WT (64 proteins with log_2_FC ≥ 2 and 35 with log_2_FC ≤-2). Strikingly, the distributions of predicted biological functions were heavily skewed for both the up- and down-regulated samples. GO enrichment analysis (**Supplementary Table S4**) showed that the most enriched group of Δ*hfq* up-regulated proteins were involved in bacterial metabolism, predominantly of amino acids and lipids (**Figure 1B** and **Supplementary Table S4**). While some amino acid metabolic genes were down-regulated in Δ*hfq*, poorly characterized proteins (mainly putative lipoproteins) represented the predominant down-regulated group (**Figure 1B** and **Supplementary Table S4**).

To explore the translational dynamics of Hfq in *P. fluorescens*, we next conducted a ribosome profiling experiment comparing the SBW25 WT and Δ*hfq* mutant strains. Our analysis revealed that Hfq controls translation of 5.7% (311 out of 5910; *p*-value ≤ 0.01 logCPM ≥ 1) of total cellular mRNAs (**Figure 1C**). Of these, 255 messengers (4.7% of all genes, 82% of the mistranslated genes) were significantly more translated in Δ*hfq* than in the WT while 56 (1% of all genes) were downregulated, consistent with a predominant function for Hfq as a translational repressor in SBW25. This finding is in agreement with both the transcript abundance data (**Figure 1A**), and with its published mechanism as an RNA chaperone that interacts with small regulatory RNAs, which mainly act to downregulate gene expression (Fröhlich and Vogel, 2009). The largest class of differentially translated genes were those involved in bacterial metabolism, with 197 *loci* (63.3% of the total misregulated mRNAs) as opposed to 11 in information storage and processing, 23 in cell processes and signaling and 80 genes with poorly characterized function (**Figure 1C**). The GO enrichment analyses of these genes revealed that the absence of Hfq mainly results in an increased translation of genes involved in membrane transport and oxidation-reduction processes (**Figure 1C** and **Supplementary Table S4**). Regulation of the dipeptide (*dpp*) transport operon is of particular interest, because of its involvement in the transport not only of dipeptide containing compounds, but also aminolaevulinic acid, haem and single amino acids (Kiely et al., 2008). Conversely, translation of genes belonging to the functional categories of extracellular transport and protein transporter activity were downregulated in Δ*hfq* (**Figure 1C** and **Supplementary Table S4**).

Intriguingly, a set of 11 genes showed strong differences in their expression between the two replicates in both the RNA-Seq and Ribo-Seq experiments (**Supplementary Table S1**) and were excluded from subsequent analyses. This is unlikely to be coincidental or related to the quality of the datasets, as the overall reproducibility between biological replicates was very high (*R* ≥ 0.9, Supplementary Figures S3, S4) for both experiments, and apart from gene *PFLU2997*, the differentially expressed genes clustered in three operons. This stochastic variation between samples is currently unexplained, but may be linked to specific RNA structural features that dictate RNA levels and partially control relative levels of gene expression, or possibly to protein-mediated feedback mechanisms of transcriptional regulation (Singh, 2011). In both cases the variability is not suppressed at the translational level. It is also possible that the expression of these genes is critically affected by the random variation exploited by genetically identical cell populations (Norman et al., 2015; Soltani et al., 2016).

To interrogate our Ribo-Seq dataset for evidence of more widespread Hfq influences, we calculated the ribosomal occupancy rate for every SBW25 mRNA, and plotted WT and Δ*hfq* values against each other (Supplementary Figure S5). The slope of the resulting scatter-plot (0.726 ± 0.009) indicated that the ribosomal-mRNA occupancy rate of Δ*hfq* was less than 73% of WT. This value only modestly increased (to 0.761 ± 0.009, Supplementary Figure S5) upon the removal of all Hfq-regulated genes from the analysis, and could not be explained by a reduction in ribosomal abundance in the mutant, as qRT-PCR of 16S rRNA abundance showed no significant difference between WT and Δ*hfq* (1.15 ± 0.20 of WT). In addition, we did not detect any substantial change in abundance for the ribosomal proteins and key translation factors detected in our proteomic dataset. This suggests a role for Hfq as a non-specific chaperone of the ribosome-mRNA interaction. The biological relevance of this remains unclear, as the change in ribosomal-mRNA occupancy in the Δ*hfq* strain has no effect on translation for the vast majority of mRNAs.

Although Hfq has been shown to bind directly to A-rich sequences in mRNAs, the predominant mode of action of Hfq *in vivo* is the regulation of targets in conjunction with non-coding RNAs (ncRNA) (Vogel and Luisi, 2011). To test if the absence of Hfq affects ncRNA abundance, we identified the ncRNA sequences in SBW25 using the database Rfam, then compared their levels in the Δ*hfq* and WT transcriptomes. Out of 87 ncRNAs identified (**Supplementary Table S2**), only 4 were present at altered levels in the mutant, suggesting that Hfq has little overall effect on ncRNA expression or abundance. For the 4 Hfq-affected ncRNAs, the biological functions of P15 and P6, the two ncRNAs overexpressed upon Hfq deletion are currently unknown. The two Δ*hfq* down-regulated ncRNAs belong to the *crcZ* subfamily of the Crc ncRNA family. CrcZ members are common throughout the *Pseudomonas* genus, and act as global regulators of carbon catabolite repression (CCR) by sequestering the RNA-binding protein Crc (Sonnleitner et al., 2009; Moreno et al., 2012; Filiatrault et al., 2013).

### Validation of the Global Datasets

**FIGURE 2:**
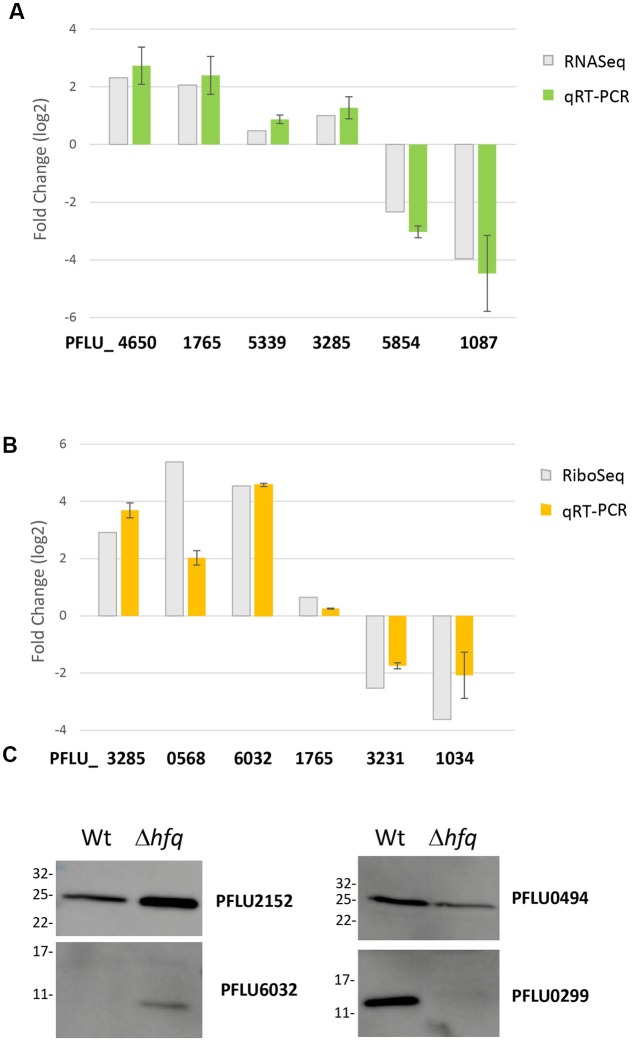
Validation of candidate loci from the global analysis datasets. **(A)** Comparative mRNA abundance data for selected loci from the *hfq* transcriptome. **(B)** Comparative mRNA abundance data for selected loci from the *hfq* translatome. In each case, log_2_ fold-change values are plotted for the *hfq* mutant versus WT SBW25. qRT-PCR values are presented alongside the corresponding fold-change observed in **(A)** the RNA-Seq experiment and **(B)** the Ribo-Seq experiment. The experiments were repeated at least twice. Data represents mean ± SD. **(C)** Western blots of selected flag-tagged proteins whose abundance changes in the Hfq proteome. The experiments were repeated at least twice. The representative blots are presented.

To validate our global regulatory data, we next conducted a series of conventional molecular biology experiments to measure mRNA and protein abundance for selected, Hfq-regulated loci. We chose a set of targets that were up- and down-regulated, or unaffected in our RNA-Seq, Ribo-Seq and iTRAQ datasets, and examined their abundance in samples grown under identical experimental conditions to those used for the original analyses. For the transcriptomic and translatomic data, qRT-PCR was used to measure mRNA abundance from total RNA (**Figure 2A**) and ribosomally associated RNA fractions (**Figure 2B**) respectively. In each case, independently obtained qRT-PCR data agreed with the results of the corresponding ‘omic experiment, strongly supporting the validity of these datasets. For the proteomic analysis, Hfq-regulated proteins were flag-tagged and expressed *in trans*. Western blotting with an anti-flag antibody was used to examine protein abundance in the WT and Δ*hfq* backgrounds (**Figure 2C**). Once again, strong agreement was observed between these data and the original iTRAQ results.

### Integration of the Three Regulatory Datasets Reveals Patterns of Global Hfq Control

**FIGURE 3:**
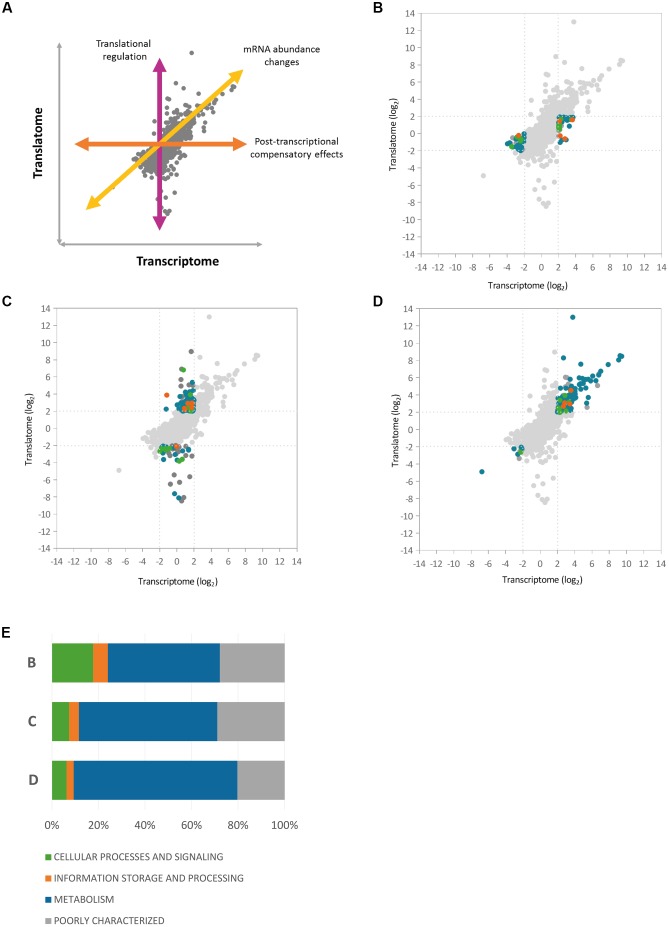
Correlation between Hfq transcriptome and translatome. Scatter-plots representing the pairwise comparisons of log_2_ ratios between Hfq transcriptome and translatome, highlighting three different regulatory classes of Hfq targets. **(A)** Illustration of the different regulatory effects on gene expression **(B)** Scatter-plot highlighting *loci* that showed altered mRNA levels in the *hfq* mutant but no corresponding change in translation **(C)** Genes showing significant translational perturbation without a comparable shift in the transcriptome. **(D)** Genes affected at both transcript abundance and translational levels. **(E)** The graph shows the relative abundance of each COG functional category at each of the regulatory levels shown in **(B–D)**.

To gain further insights into the nature of Hfq regulation in *P. fluorescens*, we next integrated the three regulatory datasets with one another (Supplementary Figure S1). To dissect Hfq control at the transcript abundance and translational levels, we first integrated the RNA-Seq and Ribo-Seq datasets. A scatter-plot representing the pairwise comparison of log_2_ ratios between the Hfq transcriptome and translatome (**Figure 3**) showed a fairly high correlation (*R* = 0.71, *n* = 5401) with a majority of data-points distributed in the middle of the plot, representing *loci* that exhibited little change in the absence of Hfq. Among the 397 *P. fluorescens loci* that exhibited significant change upon *hfq* deletion, 190 genes (47.8%) were regulated at the translational level only (3.5% of the analyzed genes), compared to 79 (19.9%) that only showed changes in steady state mRNA levels. 128 genes (32.2%) were controlled at both regulatory levels.

To analyze how functions controlled by Hfq are split between different regulatory levels, we dissected the graph into three main groups (**Figure 3** and **Supplementary Table S3**) based on the level where gene expression is regulated (**Figure 3A**). *Loci* that showed altered transcript levels in the *hfq* mutant but no corresponding change in translation included proteins from the cytochrome C family (biogenesis *loci*: *PFLU1760-PFLU1765*, *cbb3*-type cytochrome C oxidases: *PFLU4559-PFLU4561*) as well as proteins of the cytochrome bd complex (PFLU5345-PFLU5346), suggesting a role for Hfq in the control of oxidative phosphorylation. Several ABC transporter components and TonB-dependent proteins, as well as the iron scavenging protein ferredoxin, were also under transcript-level control (**Figure 3B**).

Analysis of genes showing significant translational perturbation without a comparable shift in mRNA abundance enabled the identification of mRNAs under translational control (**Figure 3C**). A large number of genes in this group encode proteins involved in amino acid and carbohydrate transport and metabolism as well as several poorly characterized proteins. Interestingly, among the Δ*hfq* down-regulated *loci* in the cellular processes and signaling subgroup, we identified genes from the general secretion pathway (type II), and *PFLU0728*, which encodes the regulatory protein RpsR from the SBW25 type III secretion system. This suggests a direct Hfq contribution to the control of *P. fluorescens* secretory pathways, as reported for several pathogens (Sittka et al., 2007; Shakhnovich et al., 2009; Schiano et al., 2010). Conversely, among the positively translationally regulated genes we found transcription factors belonging to the AsnC (PFLU2559) and AraC families (PFLU3095, PFLU4808). Changes in the abundance of these proteins (and hence altered gene transcription) could explain some of the transcript-level mis-regulation seen in the Δ*hfq* mutant.

A substantial fraction (39.8%) of the final group (**Figure 3D**); affected at both transcript abundance and translational levels, functions in the transport and metabolism of amino acids and carbohydrates. In addition, *hupA* (*PFLU6032*) encoding the DNA-binding protein HU1 was associated with increased transcript levels, and highly translated in the *hfq* mutant. It appears that Hfq not only interacts with DNA, but also cooperates in the organization of the bacterial chromosome with other proteins, including HU (Cech et al., 2016). Hfq and HU associate with the nucleoid in markedly different ways, bridging and bending the DNA respectively. Nonetheless, both Hfq and HU regulate a similar set of cellular behaviors including nucleoid structuring, recombination, transposition, growth, replication, motility, metabolism, and virulence (Phan et al., 2015). The increased abundance of HU1 may therefore represent a compensatory response to the absence of Hfq.

**FIGURE 4:**
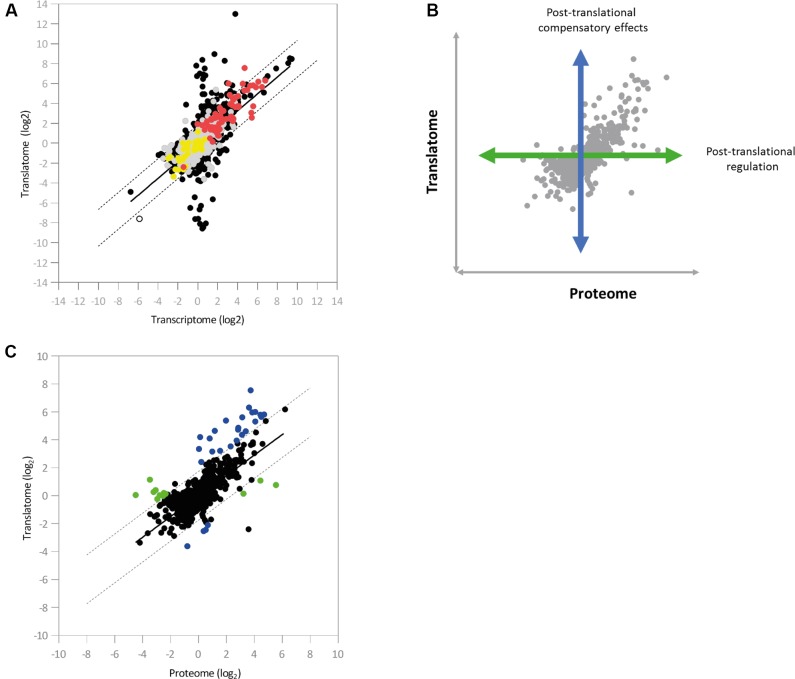
Integration of the regulatory datasets. **(A)** Color-coded integration of proteomic data into the pairwise comparison between the Hfq transcriptome and translatome (*n* = 5910). Red and yellow dots indicate *loci* that show up- and down-regulated protein abundance, respectively, in the Δ*hfq* mutant. *Loci* exhibiting no significant change in protein abundance are indicated in gray (**Supplementary Table S4**). **(B)** Illustration of the effects of post-translational control on protein abundance. **(C)** Scatter-plot showing the pairwise comparisons of log_2_ ratios between the Δ*hfq* translatome and proteome (*n* = 1867). Post-translationally regulated *loci* are marked in green, while *loci* displaying compensatory post-translational effects are marked in blue (**Supplementary Table S4**). In both cases, dashed lines indicate two-fold ratios of differential expression from the regression line (in black).

We next integrated the proteomic data into the pairwise comparison between the Hfq transcriptome and translatome. Three-dimensional representations of this data proved uninformative, so instead *loci* were colored according to the effect of *hfq* deletion on protein abundance (**Figure 4A**). We saw a strong agreement in the general direction of regulation between the proteomic and genetic datasets. Interestingly, most of the proteins detected in our experiment lay close to the diagonal regression line corresponding to transcript-level regulation (**Figure 3D**), with the vast majority lying within a two-fold ratio of differential expression on either side of the regression line.

To examine the impact of post-translational regulatory mechanisms on the Hfq proteome, we next integrated the translatomic and proteomic datasets (**Figures 4B,C**). Once again, a regression line was calculated and added to the analysis. While the majority of *loci* lay within a two-fold differential ratio of this line (i.e., translation and protein abundance are directly correlated), two substantial groups of outliers were identified. The first of these (green in **Figure 4C** and **Supplementary Table S4**) showed little translational regulation but substantially altered protein abundance, while for the second (blue in **Figure 4C** and **Supplementary Table S4**) translation significantly changes on *hfq* deletion but is not accompanied by a corresponding shift in protein abundance. In both cases, post-translational control (i.e., altered protein stability, protease activity etc.) is apparently taking place, either as a specific means of post-translational regulation or to compensate for higher-level perturbations, as we saw no evidence for increased ribosome stalling on these transcripts (Supplementary Figure S6).

## Discussion

Analyzing transcript-level regulation in isolation fails to explain much of the observed flexibility of bacterial adaptation (Arraiano et al., 2010). While there is undoubtedly a connection between protein and mRNA levels, adaptive mechanisms at the post-transcriptional and post-translational levels are also highly important, and must be accounted for if we are to produce an accurate assessment of bacterial regulation. As a core component of the global post-transcriptional networks of many bacterial species, Hfq represents a model protein to highlight the power of a multi-omics approach to gain a more accurate picture of complex regulatory pathways. By facilitating the pairing of small RNAs with their target mRNAs, Hfq affects the translation and turnover rates of specific transcripts and contributes to complex post-transcriptional networks (Vogel and Luisi, 2011). In *Pseudomonas* spp., Hfq plays a critical role during niche adaptation, with its deletion affecting phenotypes important for rhizosphere colonization of the soil-dwelling *P. fluorescens* SBW25 as well as host interactions by the related pathogens *P. aeruginosa* and *P. syringae* (Little et al., 2016).

The data we present here describe changes that arise as a consequence of *hfq* deletion in *P. fluorescens* at three different regulatory levels; mRNA abundance, protein abundance, and analysis of actively translated mRNA. Transcriptome profiling revealed that Hfq influences 212 genes, affecting in particular the transcript levels of *loci* involved in bacterial metabolism. This finding supports a major role for Hfq in the control of *P. fluorescens* metabolic versatility, in agreement with studies in other bacteria (Sonnleitner et al., 2006; Sittka et al., 2009; Torres-Quesada et al., 2010). To identify and dissect Hfq-mediated regulation at the transcript abundance and translational levels, we next performed ribosome profiling experiments. Ribo-Seq provides measurements of protein synthesis activity, reflecting both the translational status of an mRNA, and its underlying abundance (Ingolia, 2016). Our experiment revealed that Hfq negatively controls translation of 311 mRNAs. These Hfq targets mainly encode transporters and enzymes involved in amino acid and carbohydrate metabolism, as well as secretory pathway components (Type II and III), siderophore utilization and chemotaxis *loci*, and the DNA binding protein HU1. Finally, we complemented our analysis of Hfq mRNA regulation by examining the soluble Δ*hfq* proteome. Intriguingly, while this dataset confirmed the substantial Hfq regulation of amino acid and lipid metabolism seen in our RNA-based analyses, we also saw evidence of a second, Hfq up-regulated group of poorly characterized putative lipoproteins. It is currently unclear whether Hfq directly controls these proteins, or if they change abundance as an indirect response to other phenotypic changes in Δ*hfq*, perhaps as an adaptation to a sessile, aggregative morphology. The Ribo-Seq dataset also provided evidence for substantial non-regulatory chaperone activity for *P. fluorescens* Hfq, with a significantly lower level of ribosomal occupancy for mRNAs in the Δ*hfq* mutant than in WT, even once regulatory targets are excluded.

To gain further insights into the nature of Hfq regulation in *P. fluorescens* and to dissect Hfq translational regulation from transcriptional/post-transcriptional effects on mRNA abundance, we integrated the RNA-Seq and ribosome profiling datasets with one another. A direct correlation between transcript levels and translation emerged for 51% of all mRNA (*R*^2^ 0.51). For the Hfq-regulated *loci* in this group, increased/decreased mRNA abundance was matched by a corresponding increase/decrease in translational activity, with mRNA translated at a constant rate and regulation occurring at the level of transcript abundance. A second set of genes showed no change in mRNA levels but significantly altered translation. Alongside other *loci* (**Supplementary Table S3**), translational changes were seen for several amino acid uptake systems (i.e., *dpp* operon, *gltJ-I* and *livJ1*). This is supported by previous research (Pulvermacher et al., 2008; Sharma et al., 2011) that links the repression of these pathways to the Hfq-associated sRNA GcvB. Likewise, oligopeptide and dipeptide transport systems are also targets of ribo-regulatory networks in several α-proteobacteria (Torres-Quesada et al., 2010).

**FIGURE 5:**
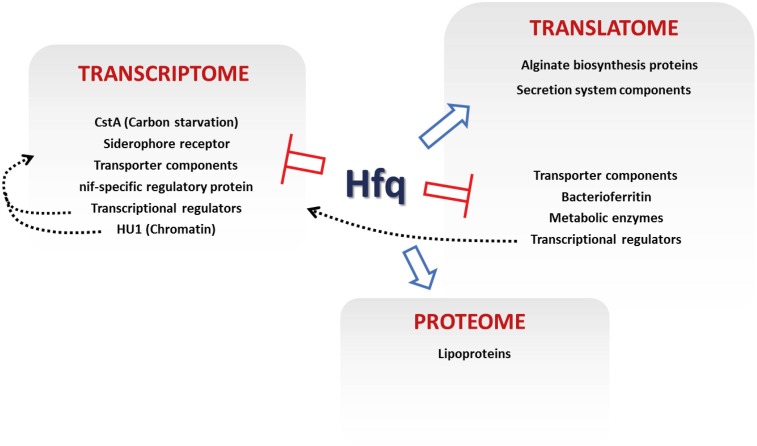
Regulatory effects of Hfq in *P. fluorescens*. The key *loci* controlled by different levels of Hfq regulation are indicated. Blue arrows show positive Hfq control, red bars denote negative control. Dashed gray arrows show proposed indirect regulations by transcriptional regulation and chromatin remodeling.

The methodology we present here does have certain limitations, primarily the inability to distinguish between direct control of specific genes by a target protein (in this case Hfq) from indirectly affected *loci* that are controlled on the same regulatory level. Nonetheless, integrating data from different global datasets as described here allows us to dissect out individual elements of complex regulatory networks (e.g., altered translational activity from transcriptional/post-transcriptional effects), and in this case reveals the influence of Hfq on a variety of cellular functions (**Figure 5**). Our integrated analysis also enables us to identify regulatory mechanisms that could otherwise be missed, or misinterpreted. For example, a third set of genes identified in the integrated analysis were those where altered transcript levels were not accompanied by a corresponding increase/decrease in translational activity. Instead, altered mRNA levels for these loci appear to be compensated for at the translational level, resulting in little overall translational perturbation compared to WT.

Strikingly, integration of the three datasets revealed that the variance in protein levels observed for the Δ*hfq* mutant closely mapped to changes in mRNA abundance (rather than translational activity). This suggests that many of the most pronounced Δ*hfq* translational targets, identified here by Ribo-Seq, are likely to have remained undetected in earlier studies. These genes code for enzymes involved in primary metabolism, transporters (i.e., porins and ABC transporter components), *katB* (PFLU 5339), and iron-uptake *loci* or targets previously not associated with Hfq-mediated regulation like PYRase (*PFLU4174*; a regulator of protein and peptide catabolism; Awadé et al., 1994). The reason for this discrepancy is likely to be methodological – quantitative proteomics is limited primarily to abundant proteins, and cannot currently attain the same level of coverage as global transcriptome studies. The integrative analysis also highlighted the presence of substantial post-translational effects upon *hfq* deletion, and allowed us to assign these effects to two broad regulatory groups. In the first, compensatory effects on protein abundance temper increased (or occasionally decreased) translational activity in the Δ*hfq* mutant. For the second group, altered mRNA translation cannot explain the substantial impacts on protein abundance we see upon *hfq* deletion. This suggests an explicit, albeit possibly indirect, Hfq effect on protein stability or production.

By integrating multiple regulatory datasets, we have been able to dissect and examine individual elements of this complex web of intracellular signaling, and to obtain several new insights into Hfq regulation in *P. fluorescens*. This integrated approach to data analysis has substantial promise for future research into bacterial adaptation to external challenges.

## Materials and Methods

### Bacterial Strains, Growth Conditions and Molecular Biology Procedures

Strains and plasmids are listed in **Supplementary Table S5**. Bacterial cultures were grown at 28°C in defined M9 medium supplemented with 0.4% pyruvate and 0.4% casamino acids (M9 pyr-cas), unless otherwise stated. Tetracycline (Tet) was used at 12.5 μg/ml. For the inducible pME6032-based vectors, IPTG was added to a final concentration of 10^-4^ M. Molecular biology methods including DNA extraction, transformation, cloning, restriction digests, electrophoresis, purification of DNA fragments and sequencing were carried out according to standard protocols (Sambrook and Russel, 2001). We performed PCR reactions using GoTaq or Phusion DNA polymerase as appropriate. Oligonucleotides are listed in **Supplementary Table S6**.

### Chromosomal Flag-Tagging of *hfq*

PCR fragments amplified with primers 1–2 and 3–4 from plasmid pSUB11 (Uzzau et al., 2001) were used to produce a C-terminal *hfq* fusion. The resulting *hfq*-flag fragment was ligated between the *Nde*I and *Xba*I sites of pME3087 (Little et al., 2016) containing the *hfq* downstream flanking region previously amplified with primers 5–6. A SBW25 WT strain with the flag-tagged chromosomal allele of *hfq* (SBW25-*hfq*::FLAG) was constructed according to the allelic exchange procedure described in (Hmelo et al., 2015).

### Flag-Tagging of Candidate SBW25 Genes

C-terminal flag-tagged versions of PFLU2152, PFLU6032, PFLU0299 and PFLU0494 were produced by amplifying each gene and its upstream regulatory region with primers 17–18, 19–20, 21–22, and 23–24 respectively, from SBW25 chromosomal DNA. The resulting PCR fragments were ligated between *BamHI* and *KpnI* sites of the pME6032 plasmid (Heeb et al., 2002), in frame with the flag sequence previously amplified with primers 15–16 from plasmid pSUB11 (Uzzau et al., 2001) and cloned between the *KpnI* and *XhoI* sites.

### RNA Extraction

Total RNA was extracted from 50 ml cultures of SBW25 WT, SBW25-*hfq*::FLAG and the Δ*hfq* mutant strain grown in M9 pyr-cas medium to the indicated OD_600_. Thirty milliliter of 60% RNAlater (in PBS) was added to each tube, and sealed tubes were vortexed and centrifuged for 10 min at 4°C. Pellets were resuspended in PBS + chilled β-mercaptoethanol RT solution, and lysed by mechanical disruption. Finally, we purified RNA from the lysate by column capture using an RNeasy Mini Kit (Qiagen). Purified RNA was subjected to additional DNase treatment (Turbo^TM^ DNase, Ambion), and RNA quantification performed with an ND-1000 Spectrophotometer.

### Isolation of Ribosome-Protected mRNAs

SBW25 and SBW25 *Δhfq* cells were cultured to late exponential phase in defined M9 medium supplemented with 0.4% pyruvate and 0.4% casamino acids. Cultures were then chilled rapidly after adding 1 mM of chloramphenicol, and harvested by centrifugation (20 min, 4000 *g*, 4°C). After resuspension of the pellets in 2 ml of lysis buffer [20 mM Hepes pH 7.8, 6 mM MgCl_2_, 100 mM NaCl, 1 mM PMSF, 16% (w/v) sucrose], cells were disrupted in a French press at 13,800 psi and the supernatant was recovered by centrifugation at 30,000 *g* for 30 min. The crude lysate was gently layered over a 35% sucrose cushion and centrifuged (2 h, 50000 RCF, 4°C). All non-ribosomal debris were removed by layering the re-suspended pellet over a second 35% sucrose cushion. RNA isolation with TRI Reagent (Sigma, T9424) was followed by DNase I treatment.

### Quantitative Real-Time PCR (qRT-PCR)

cDNA synthesis was performed as previously described (Little et al., 2016). We performed qRT- PCR using a 20 μl reaction mix containing 1 μl cDNA. At least three wells were run for each sample. Relative quantification was used to compare the abundance of candidate mRNAs in equivalent WT and SBW25-*hfq*::FLAG or SBW25 *Δhfq* samples. In each case, the abundance of each gene transcript was normalized to the WT reference sample. For the 2-ΔΔC_t_ method (Livak and Schmittgen, 2001; Bustin et al., 2009), results were presented as n-fold increase relative to the reference sample. The ΔCt-values were examined using the Student’s *t* test to determine whether datasets for relative gene expression were significantly different from those in a chosen calibrator. Primers were experimentally validated for suitability to the 2-ΔΔC_t_ method, and are listed in **Supplementary Table S6**. We used melting curve analysis to confirm the production of a specific single product from each primer pair. Each experiment was repeated at least twice independently.

### Immunoblot Analysis

Protein concentrations of lysate supernatants were estimated via an A_280_ measurement, and gels/blots were normalized by loading equal amounts of total protein per well (this was subsequently validated by Coomassie Blue staining). In the case of the C-terminal flag tagged Hfq protein, samples were normalized following comparison of optical density for the initial cell samples. Samples were separated on 15% Tris-HCl gels, then blotted onto polyvinylidene difluoride (PVDF) membranes (Millipore). Membrane was incubated overnight in blocking solution (1X PBS pH 7.4, 0.01% Tween20, 5% milk powder), then protein was detected with 1/5000 ANTI-FLAG antibody (Sigma) and 1/6,000 anti-rabbit secondary antibody (Sigma). Bound antibody was visualized using ECL chemiluminescent detection reagent (GE Healthcare).

### RNA-Seq

SBW25 WT and Δ*hfq* cultures were grown at 28°C in M9 pyr-cas medium to the late exponential phase. RNA was then extracted as reported in the ‘RNA extraction’ section, and treated with the Ribo-Zero rRNA Removal Kit (Bacteria) (Illumina) to remove ribosomal RNA. RNA libraries were prepared using the TruSeq Stranded mRNA Library Prep Kit (Illumina), and deep sequenced by Illumina NextSeq500 Sequencing.

### RNA-Seq Data Analysis

Paired end reads were aligned to the *P. fluorescens* SBW25 reference genome (Genbank accession number NC_012660) using Bowtie2 version 2.2.9 (Langmead and Salzberg, 2012). All libraries had an overall alignment rate of over 98 percent. The resulting SAM files were processed using Perl scripts to calculate coverage at each nucleotide position of the genome and to arrive at two column text files containing counts of reads mapping to each gene in the SBW25 genome. These files were used for the calculation of FPKM values for each gene and also as input for differential gene expression analysis using Bioconductor package edgeR, according to the procedure described in the edgeR user guide. Briefly, the data was read in using the readDGE function and after the estimation of common and tagwise dispersions, the function exactTest was used to carry out pair-wise comparisons (Robinson and Smyth, 2008). Finally, the function topTags (Benjamini and Hochberg method) was used to output a table of genes with their log fold-changes and associated false discovery rates. The limma function plotMDS was used to make the PCA plots.

### Searching for ncRNAs in the SBW25 Genome

The Rfam database version 12.1 was downloaded from the EBI FTP site. The program cmscan from the Infernal package was used to search the SBW25 genome for the covariance models in the Rfam database. The output produced by cmscan was used to make a bed file for viewing in IGV/IGB.

### Ribosomal Profiling

SBW25 WT and Δ*hfq* cultures were grown at 28°C in M9 pyr-cas medium to the late exponential phase. Cells were harvested by rapid filtration as described in Oh et al. (2011). Collected cells were flash frozen in liquid nitrogen and cryogenically pulverized by mixer milling (Retsch). Pulverized cells were thawed and clarified by centrifugation. Resulting lysates were digested with MNase, quenched with EGTA and resolved by sucrose density gradient ultracentrifugation. Ribosome-protected mRNA footprints were processed as previously described (Oh et al., 2011; Becker et al., 2013) and sequenced by Illumina HiSeq2000.

### Ribo-Seq Data Analysis

Reads in fastq files received from the sequencing contractor were adaptor trimmed using a Perl script which implemented the procedure described in (Becker et al., 2013). Ribosomal RNA sequences were filtered out of the trimmed reads by aligning them against a Bowtie2 index containing only the ribosomal RNAs of SBW25. Reads not aligning to the ribosomal RNAs were then aligned to the genomic sequence of SBW25 to get SAM files. The SAM files were then used to calculate the center-weighted coverage for at each nucleotide position of the genome. For this, a Perl script was used to select alignments that were between 23 and 41 nucleotides in length and counted for nucleotide positions after trimming 11 nucleotide positions from either end of the alignment. This was done separately for reads aligning to the forward and reverse strands of the genome and the center-weighted coverage was stored in separate files for the two strands. Another Perl script was used to calculate the RPKM values for each gene based on the strand specific center-weighted coverages along the genome. The limma function plotMDS was used to make the PCA plots.

### Translation Efficiency

Read counts for each gene were normalized to gene lengths for both the Ribo-Seq and RNA-Seq data and then scaled to the same totals between the WT and Hfq. Translation efficiency was calculated as Ribo-Seq read counts divided by the RNA-Seq read counts. Finally, the fold change in translation efficiency of each gene was calculated between the WT and Hfq.

### Ribosome Stalling

Coverage at each nucleotide position for both RNA-Seq and Ribo-Seq were normalized for their sequencing depths. Then Ribo-Seq coverage for each gene was normalized to the median of the RNA-Seq coverage for the corresponding genes. Then, the maximum and average Ribo-Seq coverage, and the ratio between the maximum and average were calculated. These ratios were compared between the WT and Δ*hfq* to see if any genes showed altered stalling.

### Quantitative Analysis Using Isobaric Labeling (iTRAQ)

50 ml SBW25 WT and Δ*hfq* cultures were grown in M9 pyr-cas medium to late exponential phase at 28°C. Cellular activity was then frozen by addition of 30 ml of RNAlater [saturated (NH_4_)_2_SO_4_, 16.7 mM Na-Citrate, 13.3 mM EDTA, pH 5.2] containing protease inhibitors. Cells were pelleted by centrifugation and washed three times with 10 mM HEPES pH 8.0 + protease inhibitors, before re-suspension to a final volume of 200 μL. Seven hundred microliter pre-cooled RLT + β-mercaptoethanol buffer (RNeasy Mini Kit, QIAGEN) was added and samples lysed with two 30 s Ribolyser pulses at speed 6.5. Supernatant was removed, and the soluble fraction separated by ultracentrifugation (279,000 *g*, 30 min, 4°C). After determination of protein concentration, the soluble proteins were precipitated with chloroform-methanol. Two biological replicates of wildtype and mutant samples were analyzed in one iTRAQ 4-plex experiment. Specifically, aliquots of 100 μg of protein were dissolved in 5% sodium deoxycholate (SDC), 20 mM sodium phosphate buffer pH 8, reduced and alkylated, and trypsin digested with 1% SDC final concentration. After SDC removal and concentration, the samples were labeled with iTRAQ tags according to the manufacturer’s instructions (AB Sciex, Framingham, MA, United States). Labeled samples were mixed, desalted on a C18 SepPak column (Waters Ltd, Manchester, United Kingdom) and fractionated by high-pH reversed phase chromatography on an XBridge^TM^ Peptide BEH C18 column, 4.6 × 250 mm (Waters) generating 14 fractions. The fractions were then analyzed by nanoLC-MS/MS on an Orbitrap Fusion^TM^ Tribrid^TM^ Mass Spectrometer coupled to an UltiMate^®^ 3000 RSLCnano LC system (Thermo Fisher, Waltham, MA, United States). Aliquots of the re-dissolved peptides were loaded and trapped using a pre-column which was then switched in-line to an analytical column (Acclaim PepMap C18, 2 μm, 75 μm × 250 mm, Thermo) for separation. Peptides were eluted with a main gradient of 6–36% acetonitrile in water/0.1% formic acid in 74 min at a flow rate of 0.3 μl min-1. The column was connected to a 10 μm SilicaTip^TM^ nanospray emitter (New Objective, Woburn, MA, United States) for infusion into the mass spectrometer. The acquisition was performed using a multi-notch MS3 reporter ion method (McAlister et al., 2014). The following acquisition parameters were used: MS1 (precursor): orbitrap resolution 60k, Scan Range (m/z) = 400-1600 (quadrupole), AGC Target = 2e^5^, DataType = Profile; MS2 with CID in IT: Top 10, threshold 2e^4^, AGC Target = 5e^3^, Collision Energy (%) = 30, dynamic exclusion 60 s, DataType = Centroid; MS3 Multi-notch Isolation (SPS): Number of Notches = 10, ActivationType = HCD, Collision Energy (%) = 55, MS2 Isolation Window = 2.5, Orbitrap Resolution = 30K, AGC Target = 7e^4^.

### Protein Quantification and Statistical Analysis

The set of 14 raw files was processed for reporter ion quantification using Proteome Discoverer 2.1.1.21 (Thermo) with Mascot 2.4.1 (Matrixscience, London, United Kingdom) as the search engine. The database search was performed on the protein sequences of *Pseudomonas fluorescens* SBW25 downloaded from Uniprot.org (6388 sequences) and the MaxQuant contaminants database (249 sequences^1^) with trypsin as protease, 2 missed cleavages, 6 ppm precursor and 0.6 Da fragment tolerance and a minimum peptide length of 6 amino acids. Carbamidomethylation of cysteine was used as static modification, oxidation (M) and deamidation (N,Q), as variable modifications. Strict FDR was set to 0.01 in Percolator. Reporter ion intensities were calculated as intensities from the MS3 spectra with 20 ppm tolerance using the most confident centroid. Reporter ion values were corrected for isotopic impurities using the manufacturer provided factors. Missing values were replaced with the minimum value. The PSM table was exported from Protein Discoverer and filtered for peptides unique to protein groups with a co-isolation threshold of ≤ 30%, an average S/N ratio of ≥ 3 and a *q*-value of ≤ 0.01. The values were transformed to log_2_ and quantile normalized using the normalizeQuantiles function in the BioConductor package limma (Ritchie et al., 2015). Log_2_ ratios were calculated for reporter ion values 116 and 114 (bioreplicate 1) and 117 and 115 values (bioreplicate 2). The mean of the PSM reporter ion abundances ratio was calculated for each protein (minimum 3 abundance counts). The resultant two sets of ratios calculated for all peptides of a given protein were subjected to a paired Wilcox test. The same procedure was applied to all proteins to obtain the corresponding *P*-values. *P*-values were adjusted for multiple testing by applying the Bonferroni method in the p.adjust function in R (Signal *P*-values in **Supplementary Table S1**). After filtering proteins for signal quality, the variability between the samples was taken into account to identify the significantly regulated protein. A biological sample *P*-value was calculated by making a dataframe in R, in which the rows correspond to proteins filtered on the basis of the signal *P*-value and two columns corresponding to ratio 116/114 and ratio 117/115. The limma function lmFit was used to fit a linear model to each gene treating the two columns as biological replicates. The limma function ebayes was used to get *P*-values which were then adjusted by the function p.adjust using the Benjamini and Hochberg method (Biological sample *P*-values in **Supplementary Table S1**). Finally, to apply a protein level identification filter to the data, the FDR was calculated by the Protein FDR Validator Node in Proteome Discoverer and considered to select only those proteins with high confidence (1% FDR).

### Integrative Data Analysis

RNA-Seq, Ribo-Seq and iTRAQ data sets were combined and viewed in as described in the manuscript, using bespoke Perl and R scripts and by using R interactively.

## Data Availability

Sequencing data is available in the ArrayExpress database (www.ebi.ac.uk/arrayexpress) under accession numbers E-MTAB-5396 (RNA-Seq) and E-MTAB-5408 (Ribo-Seq). The mass spectrometry proteomics data have been deposited to the ProteomeXchange Consortium via the PRIDE (Vizcaíno et al., 2016) partner repository with the dataset identifier PXD005621.

## Author Contributions

Experiments were conceived and designed by LG and JM. LG performed the experiments and together with GC carried out the computational data analysis. GS performed the iTRAQ quantitative mass spectrometry. CG and GK contributed expertise/materials/analysis tool and critically reviewed the manuscript. LG and JM wrote the manuscript. All authors: final approval; agreement for accountability.

## Conflict of Interest Statement

The authors declare that the research was conducted in the absence of any commercial or financial relationships that could be construed as a potential conflict of interest.

## Footnotes

1 www.coxdocs.org

## Supplementary Material

TABLE S1 Parallel global analyses of the *P. fluorescens* hfq null mutant.

TABLE S2 ncRNAs analysis.

TABLE S3 Correlation between Hfq transcriptome and translatome.

TABLE S4 Integration of the regulatory datasets.

TABLE S5 Strains and plasmids.

TABLE S6 Oligonucleotides.
