# Supplementary material for: Analyzing the Complex Regulatory Landscape of Hfq – an Integrative, Multi-Omics Approach
Source: Front Microbiol. 2017 Sep 20;8:1784. doi: 10.3389/fmicb.2017.01784 (PMC5627042; doi:10.3389/fmicb.2017.01784)
Supplement: TABLE S5 — Strains and plasmids. [file Table_5.docx]

**Table S5.** Strains and plasmids

| **Strains** | **Description** | **Reference** |
| --- | --- | --- |
| ***Pseudomonas*** |  |  |
| SBW25 | Environmental *P. fluorescens* isolate | (Rainey and Bailey, 1996) |
| SBW25 ∆*hfq* | SBW25 with *hfq*(*PFLU_0520*) deleted | This study |
| ***E. coli*** |  |  |
| DH5α | *endA*1, *hsdR*17(r_K_-m_K_+), *supE*44, *recA*1, *gyrA* (Nal^r^), *relA*1, Δ(*lacIZYA-argF*)U169, *deoR*, Φ80*dlacΔ(lacZ)M15* | (Woodcock et al., 1989) |
| **Plasmids** |  |  |
| pSUB11 | Amplification vector for *flag-*FRT-Kan^R^-FRT cassette | (Uzzau et al., 2001) |
| pME3087 | Tet^R^, suicide vector; ColE1-replicon, IncP-1, Mob | (Voisard et al., 1994) |
| pTS-1 | pME3087 derivative containing a *sacB* counter-selection marker | Lab collection |
| pTS-1*hfq*FLAG-*hflx* | pTS-1 with *hfq* Flag-tagged allele as *NdeI-Xba*I fragment and the *hfq* downstream flanking region (*hflX*) as *Xba*I-*Bam*HI fragment | This study |
| pME6032 | Tet^R^, pVS1, IPTG-inducible shuttle expression vector | (Heeb et al., 2002) |
| pME6032*hfq* | pME6032 derivative with the *hfq* gene as *EcoRI*- *XhoI* fragment | This study |
| pME6032-*flag* | pME6032 derivative with the *flag* sequence as *KpnI*- *XhoI* fragment | This study |
| pME6032-pr*PFLU2152flag* | pME6032-*flag* derivative with the *PFLU2152* and its upstream regulatory region as *BamHI*- *KpnI* fragment | This study |
| pME6032-pr*PFLU6032flag* | pME6032-*flag* derivative with the *PFLU6032* and its upstream regulatory region as *BamHI*- *KpnI* fragment | This study |
| pME6032-pr*PFLU0299flag* | pME6032-*flag* derivative with the *PFLU0299* and its upstream regulatory region as *BamHI*- *KpnI* fragment | This study |
| pME6032-pr*PFLU0494flag* | pME6032-*flag* derivative with the *PFLU0494* and its upstream regulatory region as *BamHI*- *KpnI* fragment | This study |
