# Supplementary material for: Analyzing the Complex Regulatory Landscape of Hfq – an Integrative, Multi-Omics Approach
Source: Front Microbiol. 2017 Sep 20;8:1784. doi: 10.3389/fmicb.2017.01784 (PMC5627042; doi:10.3389/fmicb.2017.01784)
Supplement: TABLE S6 — Oligonucleotides. [file Table_6.docx]

**Table S6.** Oligonucleotides

|  | **Name** | **Sequence (5’ 🡪 3’)** |
| --- | --- | --- |
| 1 | hfq NdeI F | GGAATTCCATATGATGTCAAAAGGGCATTCGC |
| 2 | hfq 258 SalI R | GCGTCGACGGCGTTACCTGGCTCAGCG |
| 3 | FLAG SalI F | GCGTCGACGACTACAAGGACCACGAC |
| 4 | FLAG XbaI R | GCTCTAGATCACTTGTCGTCGTCGTCC |
| 5 | hflX XbaI F | GCTCTAGATAGGAGTCTCCTTTGTTCT |
| 6 | hflX 258 BamHI R | CGGGATCCGATTGAAGATCACCAGATC |
| 7 | hflX qRTPCR_1 | CACGACGAAGAAGGTGTCAG |
| 8 | hflX qRTPCR_2 | CGATGAACTCTGTCGGTACC |
| 9 | hfq qRTPCR_1 | AAAACACCGTCAGCCAGATG |
| 10 | hfq qRTPCR_2 | GTCAGCTGCATCATCACCTG |
| 11 | rpoD qRTPCR_1 | CAACGAAGTAGACGAAAGCTC |
| 12 | rpoD qRTPCR_2 | GACGGTTGATGTCCTTGATCTC |
| 13 | hfq EcoRI F | CGGAATTCATGTCAAAAGGGCATTCGCT |
| 14 | hfq XhoI R | CCGCTCGAGTCAGGCGTTACCTGGCTCA |
| 15 | FLAG KpnI F | GGGGTACCGACTACAAGGACCACGAC |
| 16 | FLAG XhoI R | CCGCTCGAGTCACTTGTCGTCGTCGTCC |
| 17 | prPFLU2152 BamHI F | CGGGATCCGAGGTTGCTCCGGGCGGCG |
| 18 | PFLU2152 KpnI R | GGGGTACCtttgcgcacggtgcgctgttc |
| 19 | prPFLU6032 BamHI F | CGGGATCCATACCGGTCGTTCTGTCGG |
| 20 | PFLU6032 KpnI R | GGGGTACCcgcgttgacagtgtctttcag |
| 21 | prPFLU0299 BamHI F | CGGGATCCGTGTTAGGGCGACGATTATAGCG |
| 22 | PFLU0299 KpnI R | GGGGTACCGCGGACACGGCGCTCGGTG |
| 23 | prPFLU0494 BamHI F | CGGGATCCGTCCCATCCTCGTTTAGCCTG |
| 24 | PFLU0494 KpnI R | GGGGTACCGCGGCGCGCCGGCACCAGC |
| 25 | PFLU0494 F | TACCCCTTCCAGAAAACCCC |
| 26 | PFLU0494 R | TAGGGGATCTGCAACTCGAC |
| 27 | PFLU4650 F | CGGGATGATTCGTGGCTTG |
| 28 | PFLU4650 R | CAAGGTCGCGTCTTCAAACT |
| 29 | PFLU1765 F | AAGTGCCAGAACCAGGACAT |
| 30 | PFLU1765 R | CGATGATCTGCTGGTTGTCC |
| 31 | PFLU5854 F | GGTTGCGGCAAAAGTACCTT |
| 32 | PFLU5854 R | GTGTCTTCAATGGCTTCGCT |
| 33 | PFLU1087 F | CCCGTTTACCTCGCTGAAAC |
| 34 | PFLU1087 R | CTTCAACCCGCGCTTCATAA |
| 35 | PFLU0568 F | AACTTGACTGACGTTGCGAC |
| 36 | PFLU0568 R | TCAGCAGGCGAATGATCTCT |
| 37 | PFLU3285 F | TGATCTGACCAACCCCTTCC |
| 38 | PFLU3285 R | ATTGCTTCTTGAACGAGCGG |
| 39 | PFLU3231 F | CGAGCCTTACGAAAGCCATT |
| 40 | PFLU3231 R | CCATGATCTTGATCCGCGAC |
| 41 | PFLU1034 F | TTGGGCATGATCGAGACCAT |
| 42 | PFLU1034 R | GACCCGAATACTGGCGTAGA |
| 43 | PFLU2152 F | CGCACTGTTCGAGAAGCAAT |
| 44 | PFLU2152 R | GGTGAAGAAAGCGGGAATGC |
| 45 | PFLU6032 F | GTAAACCAGAACTCGCAGCC |
| 46 | PFLU6032 R | CGCCGGTGATTTCTTCGAG |
| 47 | PFLU0299 F | GGACCAATACCCTCAAGCCT |
| 48 | PFLU0299 R | TCATTCTTACCATTGGCCGC |
