# Supplementary figures and images for: Analyzing the Complex Regulatory Landscape of Hfq – an Integrative, Multi-Omics Approach
Source: Front Microbiol. 2017 Sep 20;8:1784. doi: 10.3389/fmicb.2017.01784 (PMC5627042; doi:10.3389/fmicb.2017.01784)

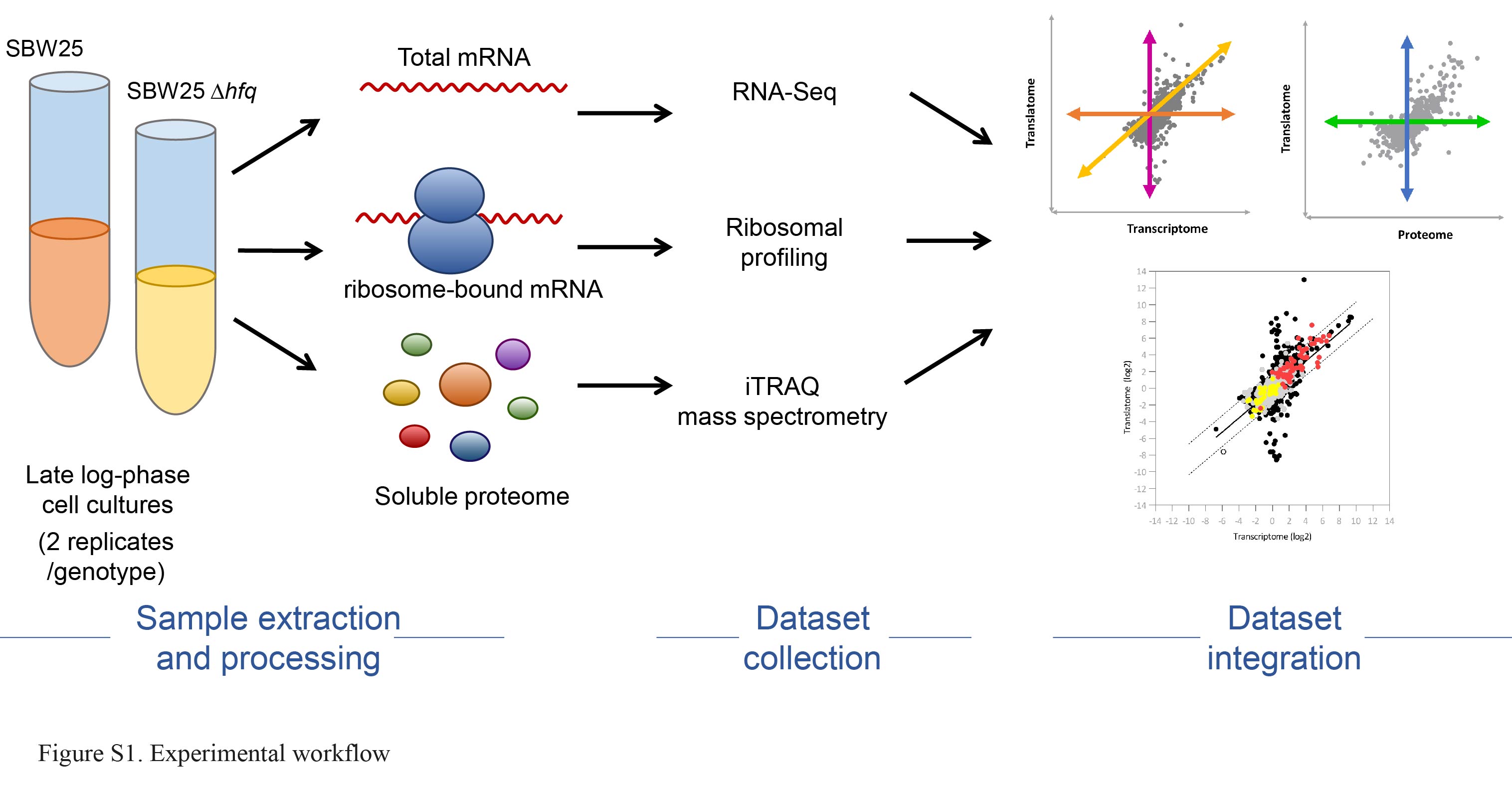

Supplement: Supplementary file 7 [file Image_1.JPEG]

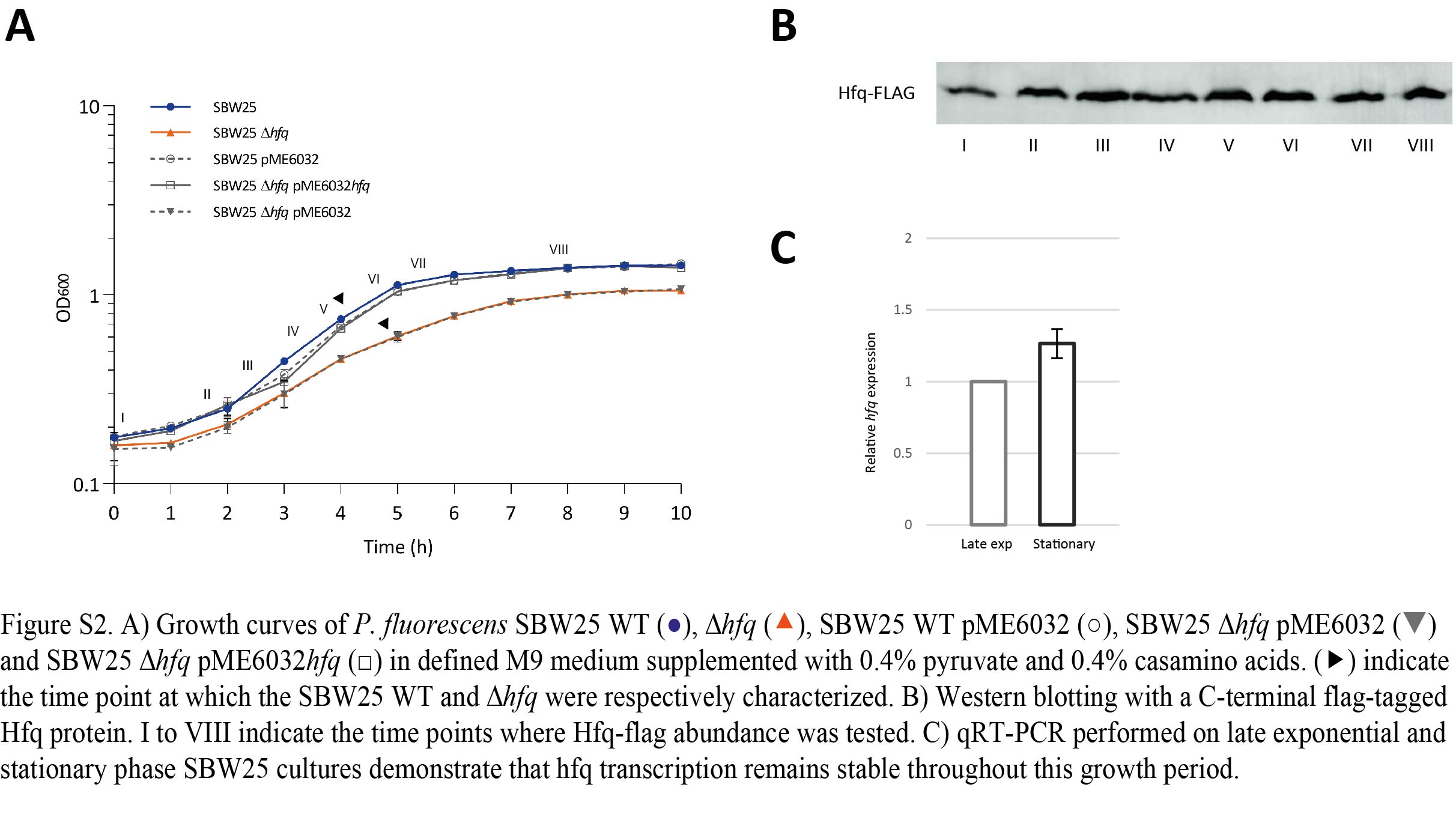

Supplement: Supplementary file 8 [file Image_2.JPEG]

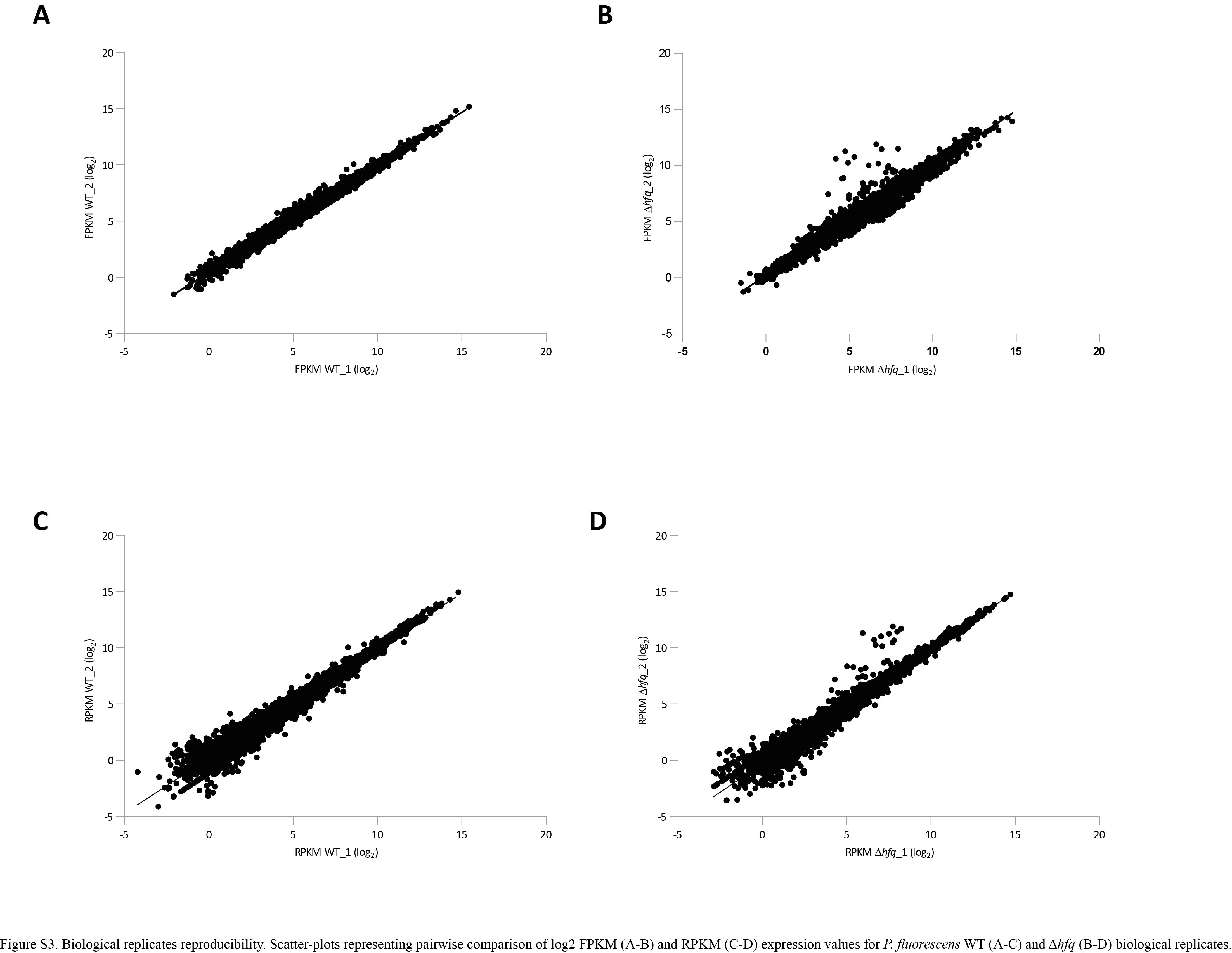

Supplement: Supplementary file 9 [file Image_3.JPEG]

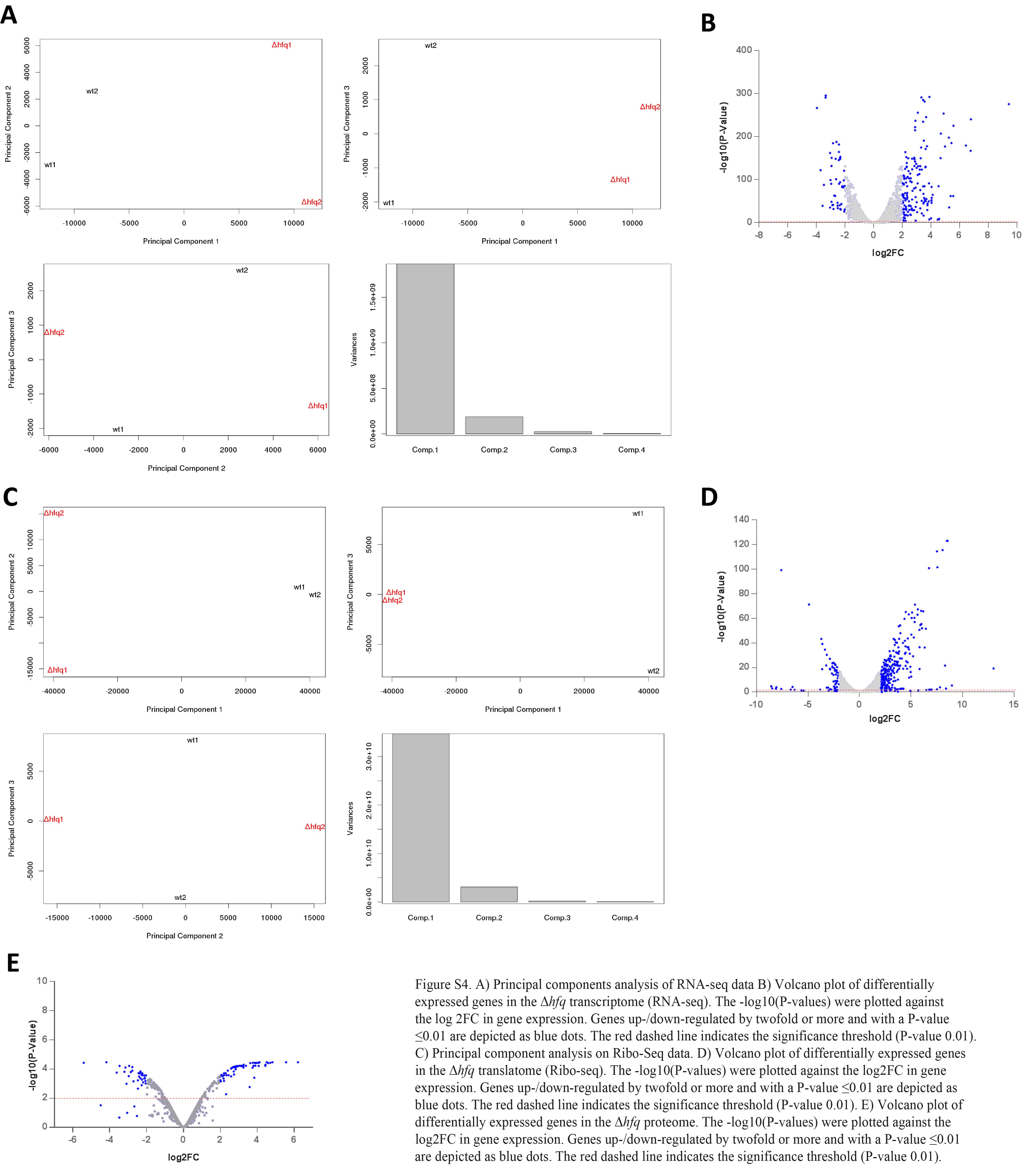

Supplement: Supplementary file 10 [file Image_4.JPEG]

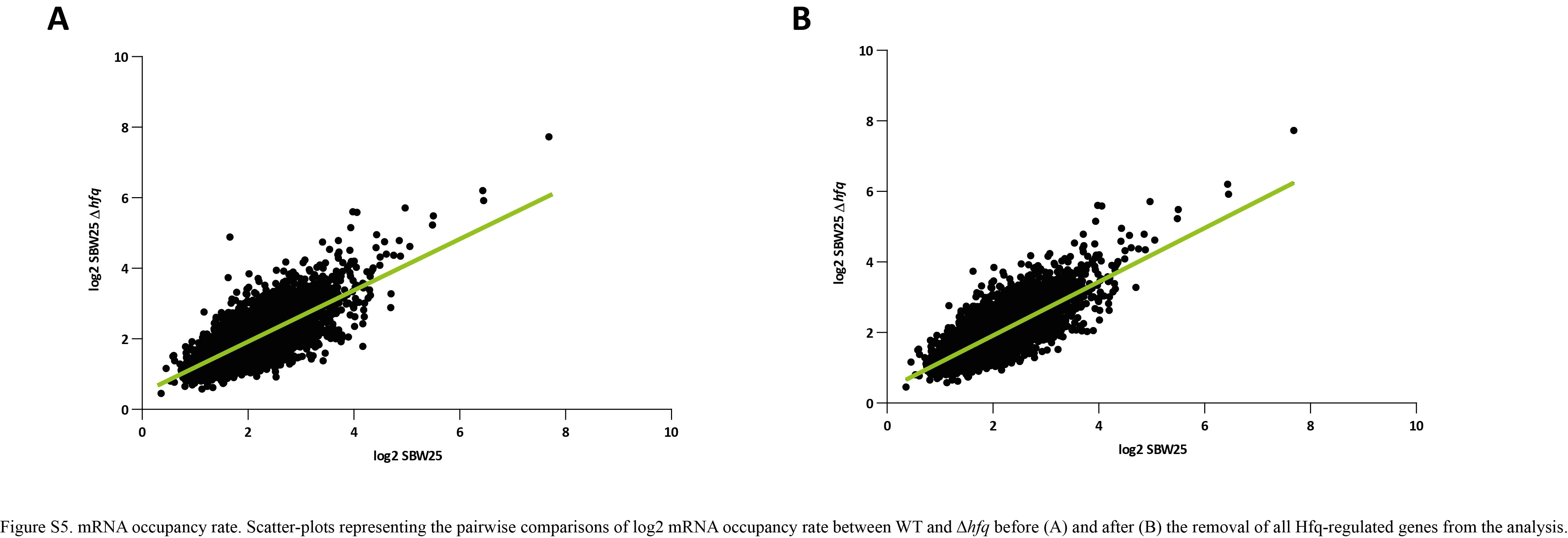

Supplement: Supplementary file 11 [file Image_5.JPEG]

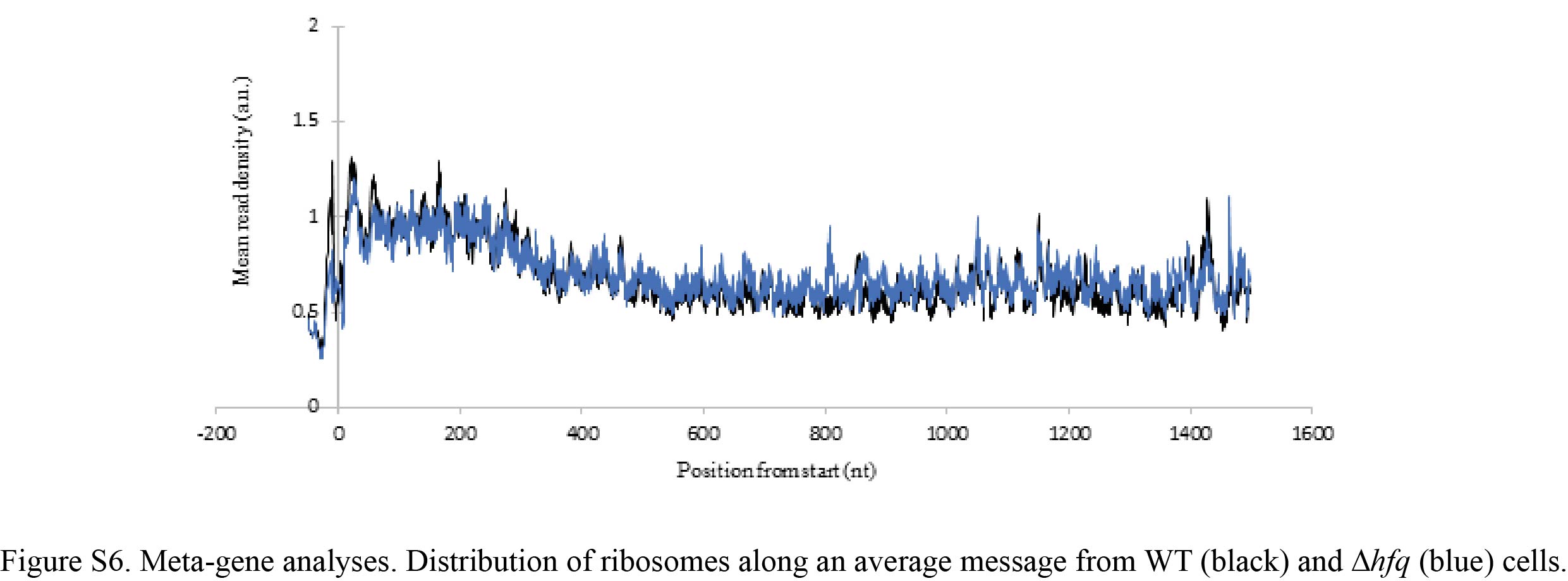

Supplement: Supplementary file 12 [file Image_6.JPEG]
